# Pre-conception and pregnancy management of bipolar disorder

**DOI:** 10.1192/j.eurpsy.2026.10285

**Published:** 2026-07-31

**Authors:** A.-L. Sutter-Dallay

**Affiliations:** BPHRC INSERM 1219-HEALTHY team, Charles Perrens Hospital and Bordeaux University, Bordeaux, France

## Abstract

**Abstract:**

Access to parenthood for patients suffering from bipolar disorder is a public health issue that must be addressed through prevention.

The preconception period is the most appropriate time to define shared prevention plans that address both the adjustment of psychotropic treatments and the most appropriate obstetric, pediatric, and psychiatric care pathways for the mother and baby.

When a patient consults while already pregnant, time constraints mean that strategies must be adapted, particularly in terms of in terms of obstetric and pediatric care.

This presentation will address the main approaches to the use of psychotropic drugs during the perinatal period, as well as the organization of coordinated and graduated integrated care pathways for mothers and newborns during the perinatal period.

**Disclosure of Interest:**

None Declared

